# Supplementary figures and images for: RhoA- and Cdc42-induced antagonistic forces underlie symmetry breaking and spindle rotation in mouse oocytes
Source: PLoS Biol. 2021 Sep 7;19(9):e3001376. doi: 10.1371/journal.pbio.3001376 (PMC8448345; doi:10.1371/journal.pbio.3001376)

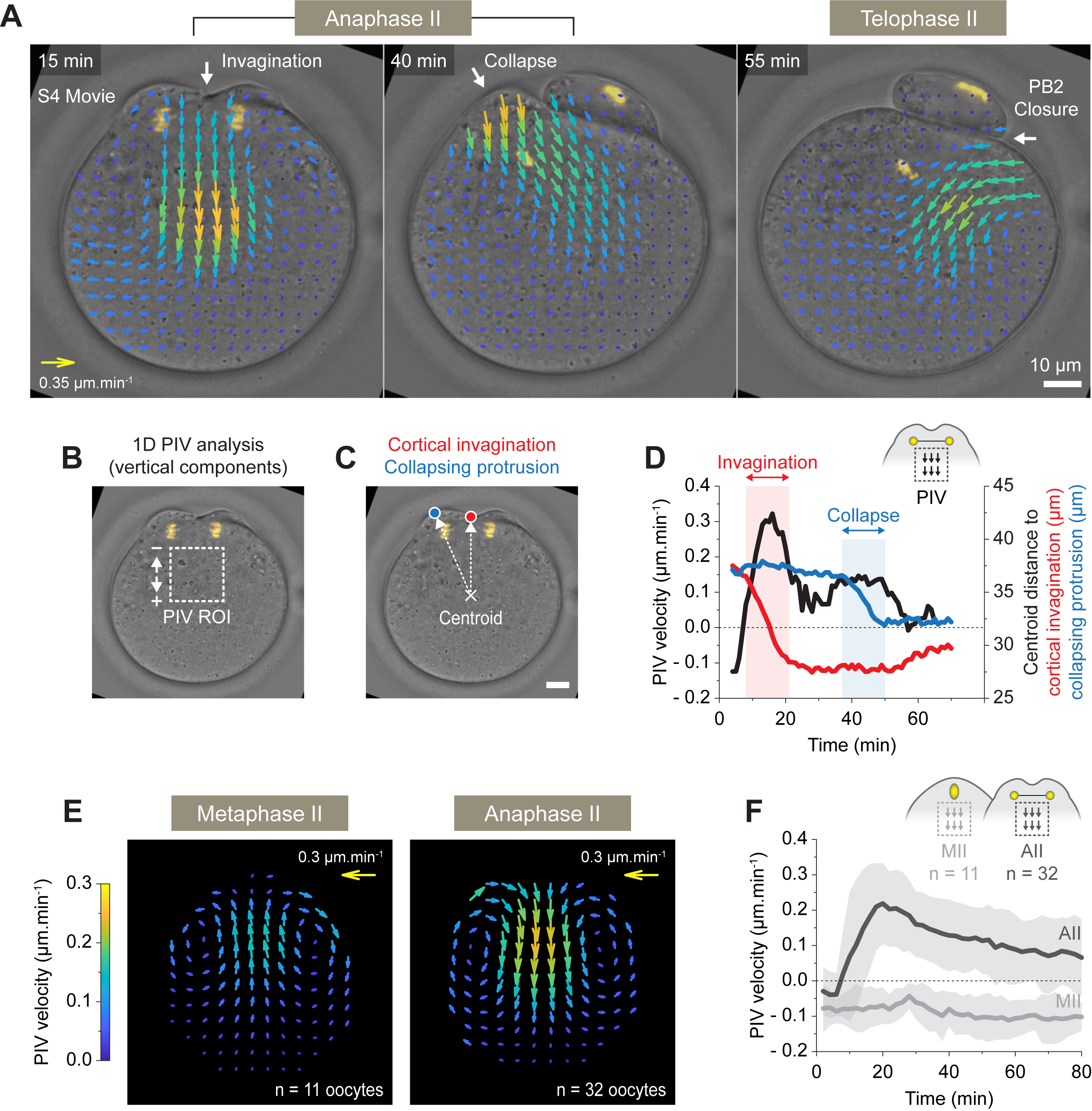

Supplement: S1 Fig — (A) PIV measurements showing cytoplasmic streaming during the course of anaphase (S4 Movie). Still images show the reverse flows induced by the cytokinetic furrow ingression (t = 15 min), the collapsing protrusion (t = 40 min), and the closure of the cytokinesis ring (t = 55 min). (B) Diagram illustrating the method used for 1D flow velocity measurements (see Methods). The dotted frame indicates the ROI for PIV measurements. Positive values indicate a flow oriented toward the oocyte center (reverse flow), while negative values indicate a flow oriented toward the polarized domain (metaphase-like flow). (C) Diagram illustrating the centroid distance to the cortical invagination (red dot) or the collapsing protrusion (blue dot). (D) Graph showing the variation over time of flow the velocity (black curve) and the centroid distance to cortical invagination (red curve) or collapsing protrusion (blue curve). (E) PIV vector fields showing the averaged cytoplasmic streaming pattern in metaphase II and anaphase II oocytes (see Methods). (F) Averaged variation over time ± SD of flow velocity in metaphase II (light gray curve) and anaphase II oocytes (dark gray curve). Measurements in E and F were performed on 11 metaphase II and 32 anaphase II oocytes, respectively, gathered from 2 and 5 independent experiments. Data underlying this figure can be found in S1 Data. Scale bars = 10 μm. PB2, second polar body; PIV, particle image velocimetry; ROI, region of interest. (TIF) [file pbio.3001376.s001.tif]

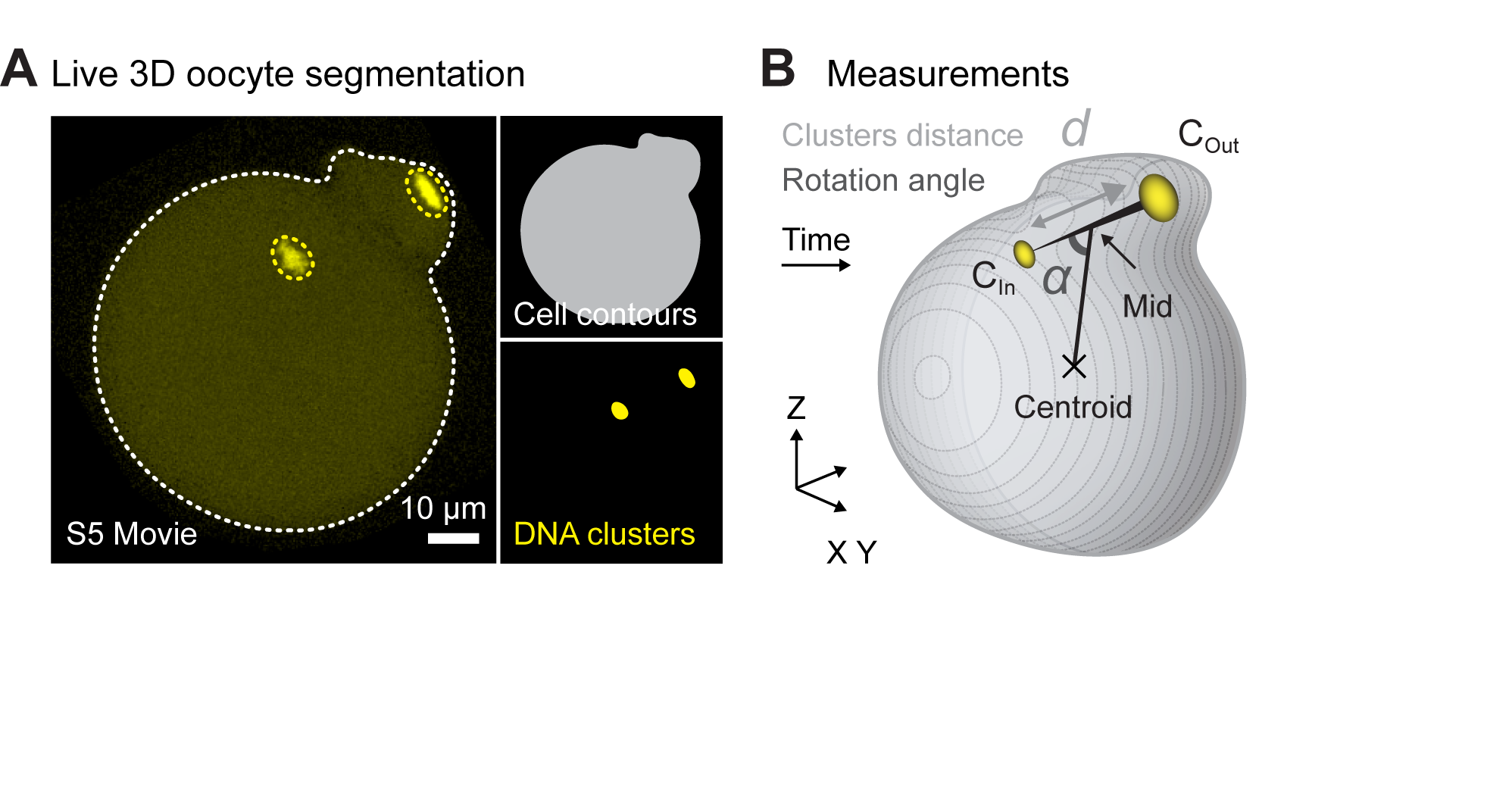

Supplement: S2 Fig — (A) Automated 3D segmentation and tracking procedure to monitor the position of the DNA clusters within the oocyte (see Methods and S5 Movie for further details). (B) Diagram showing the method used to quantify DNA cluster distance d and the spindle rotation angle α. Scale bars = 10 μm. (TIF) [file pbio.3001376.s002.tif]

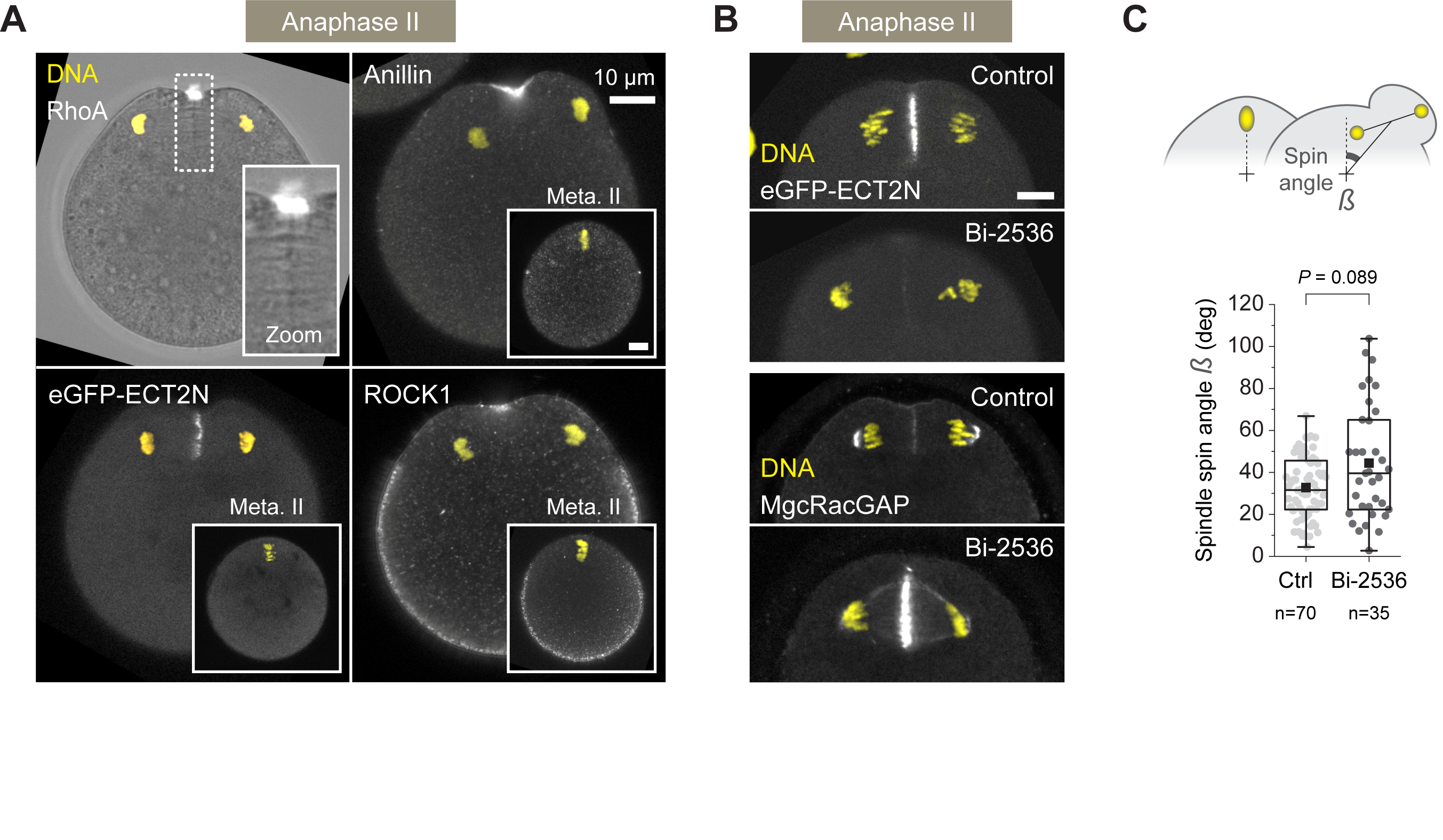

Supplement: S3 Fig — (A) Fixed RhoA, anillin, ROCK1 (immunostaining), DNA (To-Pro-3), and eGFP-ECT2N imaging of metaphase II (insets) and anaphase II oocytes (main images). Fluorescence patterns shown in A were consistently observed in a minimum of 14 oocytes gathered from a minimum of 2 independent experiments. (B) Fixed eGFP-Ect2-N and MgcRacGAP (immunostaining) imaging on control and Bi-2536–treated oocytes. The PLK1 inhibition prevented eGFP-ECT2N recruitment at the central spindle (top panels) while, in contrast, the recruitment of MgcRacGAP (immunestaining) was preserved (bottom panels). Note the enhanced MgcRacGAP signal in Bi-2536–treated oocytes, which may result from epitope unmasking, due to the release of bound ECT2. Fluorescence patterns shown in B were consistently observed in 23 oocytes (eGFP-ECT2N) and in 15 oocytes (MgcRacGAP) both gathered from 3 independent experiments. (C) Box plots showing the maximum variation of the spin angle β, as defined in top diagram and Methods, in control and Bi-2536–treated oocytes. Measurements in C were performed on 70 control oocytes gathered from 9 independent experiments and 35 Bi-2536 oocytes gathered from 6 independent experiments. Parameters for box plots and statistics in C are as described in Fig 2. p-value in C was obtained using a two-sided Mann–Whitney test. Data underlying this figure can be found in S1 Data. Scale bars = 10 μm. PLK1, Polo-like kinase 1; ROCK1, Rho-associated protein kinase 1. (TIF) [file pbio.3001376.s003.tif]

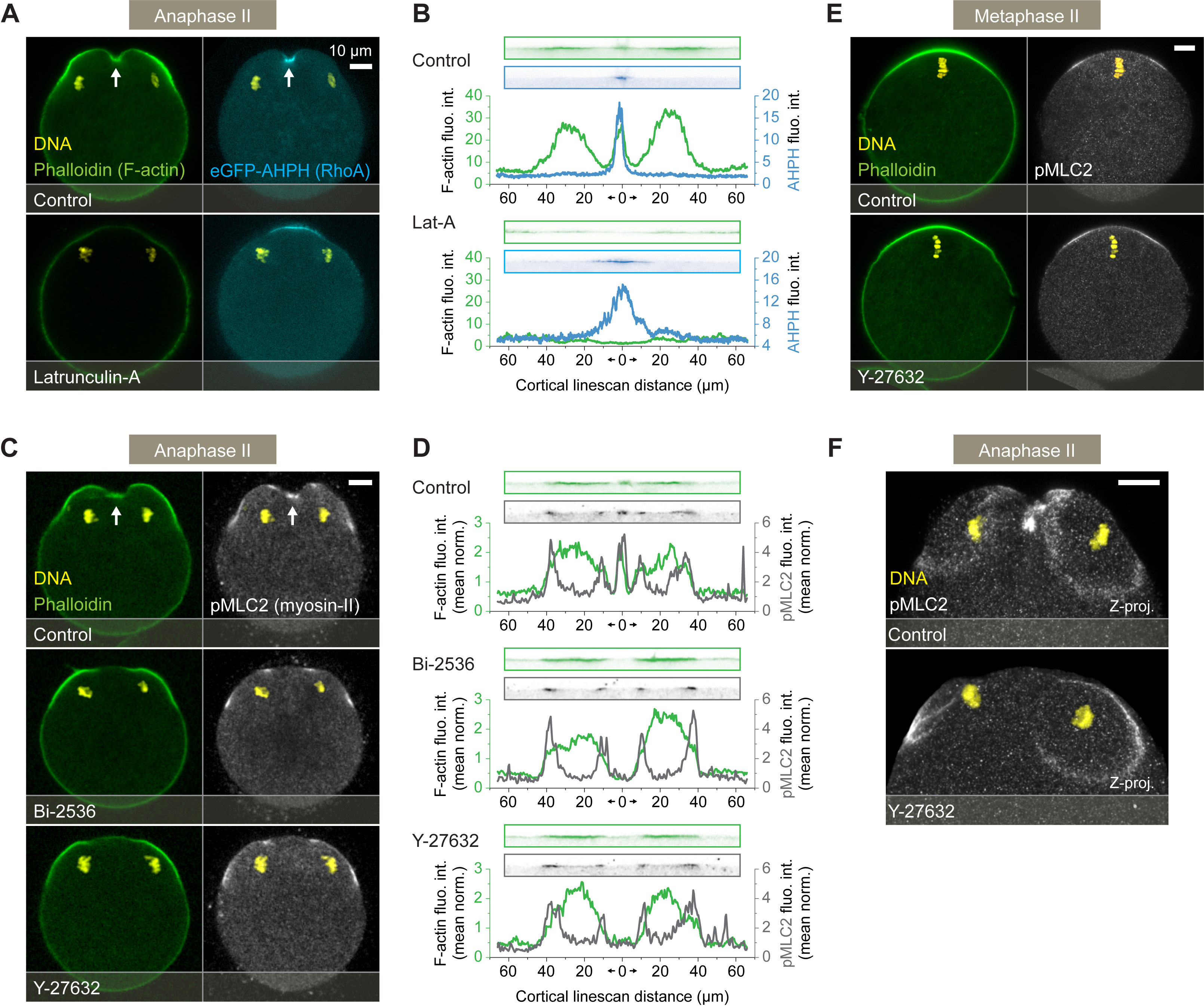

Supplement: S4 Fig — (A) Fixed F-actin (phalloidin staining), eGFP-AHPH (RhoA sensor), and DNA (To-Pro-3) imaging of control and latrunculin A–treated anaphase II oocytes. The white arrows show the F-actin and activated RhoA patch facing the central spindle. Note the persistence of RhoA activation despite the lack of F-actin accumulation upon latrunculin A treatment. Fluorescence patterns shown in A were consistently observed in a minimum of 8 oocytes gathered from a minimum of 3 independent experiments. (B) Cortical fluorescence intensity profiles recapitulating F-actin and eGFP-AHPH polarization patterns observed in A. (C) Fixed F-actin (phalloidin staining), myosin-II (pMLC2 immunostaining), and DNA (To-Pro-3) imaging of control, Bi-2536–treated (PLK1 inhibitor), and Y-27632–treated (ROCK1 inhibitor) anaphase II oocytes. The white arrows show the F-actin and myosin-II patch facing the central spindle. (D) Cortical fluorescence intensity profiles recapitulating F-actin and myosin-II polarization patterns observed in C. Note the disappearance of the central spindle-facing actomyosin patch upon PLK1 and ROCK1 inhibition. (E) Fixed F-actin (phalloidin staining) and myosin-II (pMLC2 immunostaining) imaging of control or Y-27632–treated metaphase II oocytes. (F) Fixed myosin-II (pMLC2 immunostaining) imaging of control or Y-27632–treated anaphase II oocytes. Images show maximum intensity z-projections of multiple confocal planes. Note the persistence of the F-actin cap and the myosin-II ring upon PLK1 and ROCK1 inhibition in C–F. Fluorescence patterns shown in C–F were consistently observed in a minimum of 18 oocytes gathered from a minimum of 3 independent experiments. Data underlying this figure can be found in S1 Data. Scale bars = 10 μm. F-actin, actin filament; PLK1, Polo-like kinase 1; pMLC2, phosphorylated myosin light chain 2; ROCK1, Rho-associated protein kinase 1. (TIF) [file pbio.3001376.s004.tif]

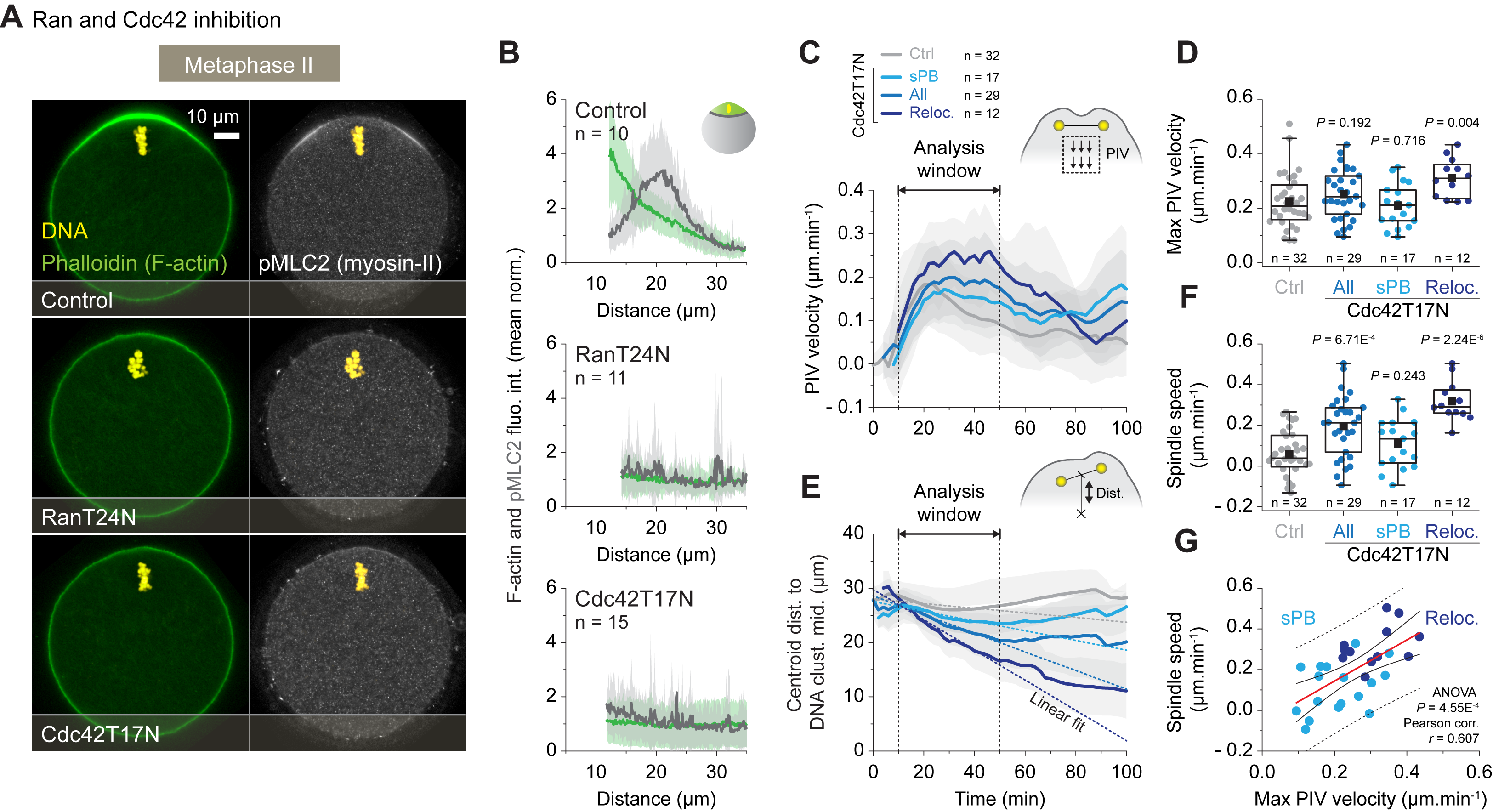

Supplement: S5 Fig — (A) Fixed F-actin (phalloidin staining), myosin-II (pMLC2 immunostaining), and DNA (To-Pro-3) imaging of metaphase II–arrested oocytes expressing dominant negative forms of the Ran and Cdc42 GTPase (respectively, RanT24N and Cdc42T17N). (B) Averaged ± SD cortical F-actin (phalloidin staining) and myosin-II (pMLC2 immunostaining) fluorescence intensities as a function of the cortex distance to DNA clusters. The fluorescence intensities are normalized to the mean of each profile. Measurements were performed on 10 controls, 11 RanT24N, and 15 Cdc42T17N metaphase II oocytes, gathered from 2 independent experiments. (C) Graph showing the averaged variation over time ± SD of flow velocity (measured as described in S1 Fig) in control (light gray) and Cdc42T17N oocytes (blue curves). (D) Box plots showing the distribution of maximum flow velocities (average of the 10% highest values) recorded within the 10- to 50-min analysis window (see C and Method). (E) Graph showing the averaged variation over time ± SD of the distance from the oocyte centroid and the DNA cluster midpoint in control (light gray) and Cdc42T17N oocytes (blue curves). The dotted lines represent linear fits performed within the 10- to 50-min analysis window. (F) Box plots showing the distribution of slopes extracted from the linear fits described in E. The slope is interpreted as the spindle relocation speed toward the oocyte centroid (see Method). (G) Scatter plot of spindle speed toward the oocyte centroid versus the maximum flow velocity. Measurements in C–G were performed on 32 control and 29 Cdc42T17N oocytes, respectively, gathered from 8 and 3 independent experiments. Parameters for box plots in D and F are as described in Fig 2. p-values (against controls) in D and F were obtained using a two-sided Mann–Whitney test. p-values in G was obtained using one-way ANOVA to test the influence of maximum flow velocities over the spindle relocation speed toward the centroid. Data underlying this figure can be [file pbio.3001376.s005.tif]

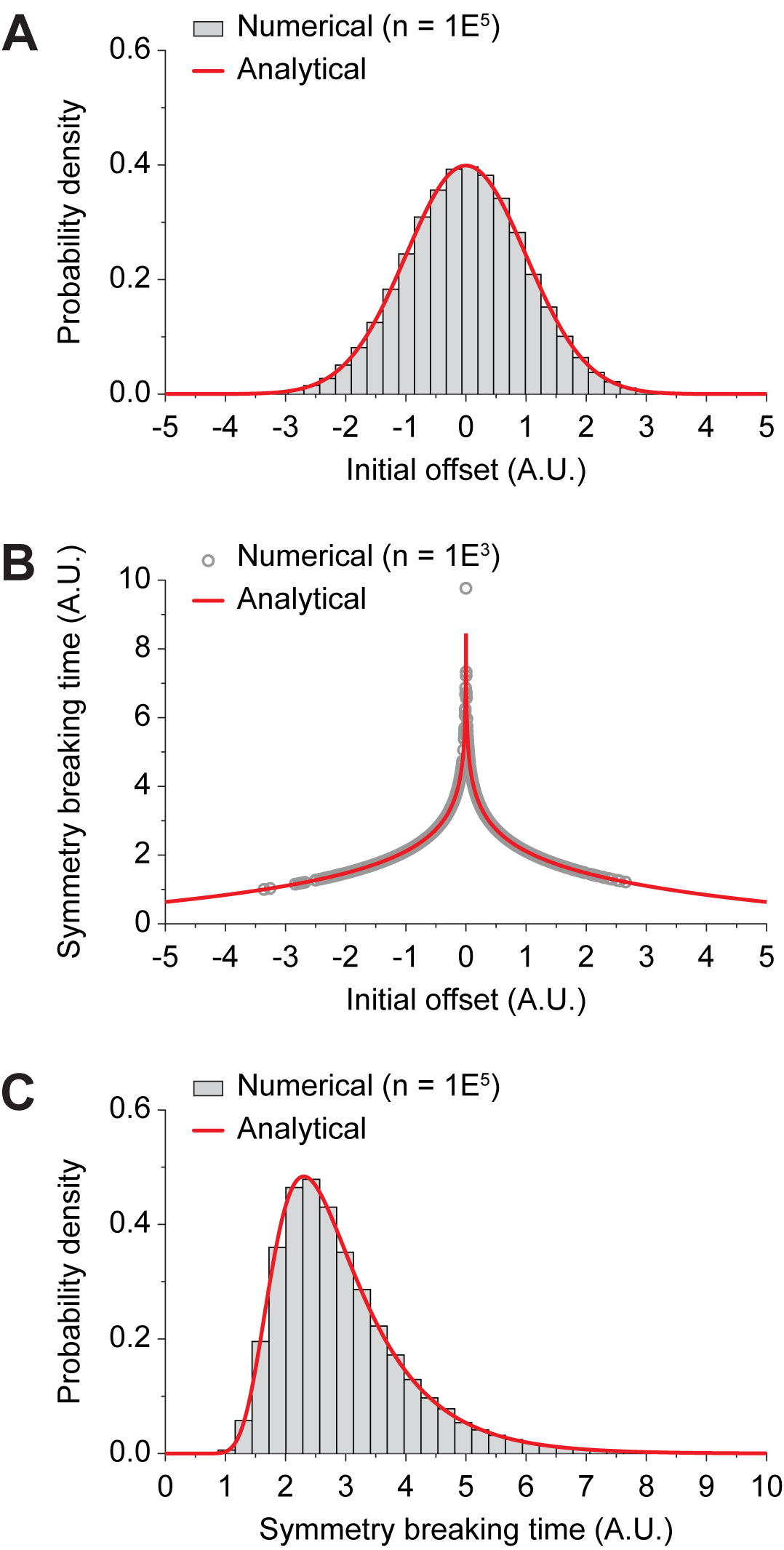

Supplement: S6 Fig — (A) Gaussian distribution of initial deviation from the unstable configuration. The red solid line shows the analytical distribution. (B) Distribution of the resulting symmetry breaking time, required to reach an arbitrary distance from the unstable configuration. The red solid line shows the analytical distribution. (C) Symmetry breaking time vs. initial offset. The red solid line shows the analytical expression. Data underlying this figure can be found in S1 Data. (TIF) [file pbio.3001376.s006.tif]
