# Supplementary material for: RhoA- and Cdc42-induced antagonistic forces underlie symmetry breaking and spindle rotation in mouse oocytes
Source: PLoS Biol. 2021 Sep 7;19(9):e3001376. doi: 10.1371/journal.pbio.3001376 (PMC8448345; doi:10.1371/journal.pbio.3001376)
Supplement: Attachment — Submitted filename: Miia_Dehapiot et al, 2021(2ndRev)_ResponseReviewers.pdf [file pbio.3001376.s021.pdf]

We are grateful to all three reviewers for their warm encouragements and suggestions for improvements. We provide below a feedback to their final comments.

Reviewer #1

The authors answered all my comments, hence I am fully satisfied with the revised work. The authors can be very proud of their work, it is a very nice and well conducted study, that I enjoyed reviewing. Congratulations to them!

We wish to thank once again Reviewer 1 for his useful comments and kind words.

Reviewer #2

The authors have done an excellent job in improving the manuscript based on previous reviewer comments. The low nocodazole experiment demonstrating that a cortical attractive force on chromosome clusters still exists during anaphase II is a nice addition. The work is a significant advance over previous work. However, there are two related issues that frustrate this reviewer and would frustrate any reader. First, the mechanism of the attractive force has not been determined. It is implied that cytoplasmic flow toward the cortex is the attractive force. The authors call this metaphase flow that persists during anaphase II. This leads to the second frustrating point. If cytoplasmic flow toward the cortex is the attractive force on both chromosome clusters, two sets of flow toward the cortex under each chromosome cluster should always be apparent in controls during anaphase II. This pair of flows toward the cortex is apparent in the averaged PIV figures (Fig. 2H and 6F) but not in the PIV figures of single movies (Fig. 1C and 5E). If the authors wish to state that metaphase flow to the cortex might be the attractive force, they should clarify how often flow toward the cortex under both chromosome clusters is observed in controls. If the authors think that metaphase flow is flow of the cytoplasm, flow cannot occur in opposite directions in the same place at the same time. If the authors think that metaphase flow is something different than the flow of the cytoplasm, they should explain this in the discussion.

We are grateful to Reviewer 2 for these positive remarks and encouragements. The low nocodazole experiments have indeed brought new evidence concerning the maintenance of cortical attraction forces during anaphase II. In this condition, we observed that DNA clusters, who eventually detached from the meiotic spindle, rapidly relocated to the overlying cortex. This was accompanied by a local flow of cytoplasmic particles, and we therefore considered that outward oriented flows, hence referred to as metaphase-like flows, could play a role in attracting DNA clusters to the cortex after the anaphase onset. However, monitoring metaphase-like flows proved challenging since the cytoplasmic streaming pattern in activated oocytes was largely dominated by inward oriented flows resulting from the cytokinetic furrow ingression. To overcome this difficulty, we prevented the cytokinetic contraction using inhibitors such as the Bi-2536 (PLK1 inhibitor) or the Y-27632 (ROCK1 inhibitor) and clearly demonstrated that metaphase-like flows still occur during anaphase II (Fig 2H and Fig 6F). Because similarly oriented flows were previously described to promote chromosomes/spindle attraction in late metaphase I and metaphase II (Yi et al, 2013; Yi et al, 2011) and because inhibiting these flows using Cdc42T17N resulted in spindle relocation to the center of the oocyte (Fig 6C and Fig 6F), we considered metaphase-like flows as a probable explanation for the maintenance of cortical attractive forces during anaphase II. Note that we acknowledge the hypothetical nature of our assertion in the

manuscript by writing that cortical attraction forces "most likely" result from metaphase-like flows (see Results line 243 and line 293).

To go further, we would like to stress that both metaphase-like and cytokinetic flows can influence the movement of cytoplasmic particles. Indeed, the isolated effect of the metaphase-like flow can be observed in Bi-2536 and Y-27632-treated oocytes (Fig 2H and Fig 6F) while the isolated effect of cytokinetic flows can be observed in Cdc42T17N-injected oocytes (Fig 6F). However, these two types of flows have different origins with, on the one hand, cytokinetic flows resulting from the pressure exerted by the invaginating cortex on the cytoplasm while, on the other hand, metaphase-like flows result from the polarized cortical flow of actin filaments (Yi et al, 2011). Consequently, the cytoplasmic streaming pattern we monitored using PIV is a composite of these two antagonistic flows (oriented in opposite direction) that necessarily interfere with each other. In our view, the metaphase-like flows are transiently masked by the stronger cytokinetic flows that arise during the peak of cytokinetic contraction. The fact that cytokinetic flows were found to be reinforced upon Cdc42 inhibition, a condition in which metaphase-like flows were prevented (see Fig 5F, S5C and S5D Fig), support this idea. In any case, more investigations will be needed to fully understand how cytoplasmic flows influence DNA cluster positioning during anaphase II. Improving the spatio-temporal resolution of confocal acquisition, as well as using more discrete tracking algorithms could probably help resolving the short-ranged metaphase-like flows throughout the second meiotic division. However, our aim here was primarily to enquire whether cortical attractive forces persisted during anaphase II, and we are pleased that this reviewer was satisfied by our new sets of data.

To answer this comment, we further elaborated on these considerations in the manuscript (see Discussion line 388-396).

Reviewer #3

The authors have done a tremendous amount of work in response to all reviewer comments and returned with a much more robust manuscript with a stronger body of evidence to support their overall conclusions. I probably would have not attempted imaging spindle rotation in IVF eggs in reponse to reviewer 2's comment 6 as this was not going to be easy from the outset, as the authors found out. Perhaps a SrCl2 activation would have done the job. Their effort is admirable nonetheless and now we at least have it on record someone tested this approach. I am overall satisfied with the revised manuscript and fully support its publication.

We are very pleased to hear these kind remarks from a leader in the field. Thank you for your encouragements and wise advice for improvements.
