# Supplementary material for: RhoA- and Cdc42-induced antagonistic forces underlie symmetry breaking and spindle rotation in mouse oocytes
Source: PLoS Biol. 2021 Sep 7;19(9):e3001376. doi: 10.1371/journal.pbio.3001376 (PMC8448345; doi:10.1371/journal.pbio.3001376)
Supplement: Attachment — Submitted filename: Miia_Dehapiot et al, 2021(Revisions)_ResponseReviewers.docx [file pbio.3001376.s020.docx]

We wish to thank the Reviewers for their constructive comments and suggestions, that allowed us to strengthen our conclusions, and improve the overall manuscript. We provide below a point-by-point answer to all comments that have been raised. On some occasions, we refer to the literature, and the relevant references are listed at the end of this document.

**Reviewer #1**

Dehapiot et al. analyze here the process of spindle rotation necessary for the extrusion of the second polar body in mouse oocytes. They provide convincing evidence that after parthenogenetic activation, the central spindle initiates furrow ingression via the classical Rho A activation pathway locally promoting cytokinetic myosin II ring formation. This initial event triggers a series of cortical reorganizations, such as actomyosin polarization above the mass of chromosomes that wins the tug of war between the two separated masses at opposite spindle poles, that will further amplify the breaking of symmetry. They also suggest that the polarized cortex exerts forces on the chromatid clusters. Eventually, they propose a very simple model with two antagonistic forces, one exerted by furrow ingression and cortical attraction, that recapitulates some features of spindle rotation.

In the whole I find the iconography presented in this manuscript to be awesome, that most of the experiments are sound and well executed and hence convincing. I would have just two minor comments.

1/ While most of the experimental evidence is convincing, the data on cortical attraction presented in Figure 5 is to me the weakest part. What exactly is the nature of this cortical attraction? Can you detect actin filaments promoted by local Arp2/3 activation connecting the mass of chromosomes or the spindle to the cortex? If you locally inhibit Arp2/3, via CK666 for example, do you also observe the spindle going away from the cortex and local disappearance of potential connections between the spindle and the cortex?

**Comment 1.1**

In response to this comment, which was also raised by the two other reviewers, we conducted a series of new experiments providing additional insights concerning these attractive forces. We present below the relevant new experiments and later discuss the possible involvement of actin filaments in connecting the mass of chromosomes or the spindle to the cortex.

Cortical attractive forces (new experiments).

We first found striking evidence supporting the existence of cortical attractive forces by treating oocytes with a low dose of nocodazole (5 µM) while they were already engaged in spindle rotation (see live nocodazole experiments in Fig 5A and S9 Movie). Indeed, by doing so, we were able to slightly disrupt the spindle integrity and occasionally triggered the detachment of DNA clusters from the meiotic apparatus. Interestingly, when the internalized cluster detached, it systematically moved toward the nearest cortical protrusion in a highly directional manner (see bottom panel in Fig 5A). This resulted in a sharp decrease of the distance between the internalized cluster and the cortex (see *dIn* in Fig 5B), which was found to be significantly reduced, as compare to controls, at the end of division (Fig 5C). These experiments showed unambiguously that DNA clusters still undergo attractive forces from the polarized cortex during anaphase II.

Next, our nocodazole experiments also suggested that these attraction forces emerge from localized cytoplasmic flows induced by the polarized cortex. Indeed, we noticed that, along with the detached DNA cluster, many cytoplasmic particles were continuously flowing toward the cortical protrusion (see manual tracking of cytoplasmic particles and PIV in Fig 5D, Fig 5E and S9 Movie). This observation, that was also made in control oocytes (see control in Fig 5E), indicates that local metaphase-like flows persist during anaphase II, despite the general reversal of cytoplasmic streaming due to the cytokinetic contraction. The persistence of these flows could further be demonstrated by inhibiting the cytokinetic contraction using PLK1 (Bi-2536) and Rock-1 (Y-27632) inhibitors. Indeed, by doing so we observed that, while the cytoplasmic streaming inversion was prevented, metaphase-like flows, oriented toward the polarized cortex, were still present under these inhibitory conditions (see Fig 2H and 6F). Note that the combined inhibition of Rock-1 and Cdc42 resulted in a complete loss of cytoplasmic streaming, indicating that metaphase-like flows emerge downstream of Ran/Cdc42 signaling (Fig 6F).

Finally, we were able to reproduce the spindle relocation phenotype observed in Cdc42T17N-injected oocytes by using the Arp2/3 inhibitor, CK-666 (Fig 6D). We also succeeded to rescue this phenotype, in both Cdc42T17N and CK-666 treated oocytes, by preventing the cytokinetic contraction with the Rock-1 inhibitor, Y-27632 (Fig 6C and Fig 6D). Taken together, these results suggest that Cdc42, through its ability to induce Arp2/3-dependent polarization of the F-actin cortex, generates attractive forces that are necessary to counteract the cytokinetic reverted flows. As described above, these forces most likely result from metaphase-like flows that persist during anaphase II.

See modifications concerning these new experiments in Results lines 227-298, new Fig 5 and Fig 6.

Involvement of actin filaments.

To provide some insights about the potential involvement of actin filaments connecting masses of chromosomes or spindle to the cortex, we imaged live oocytes expressing the eGFP-UtrCH F-Actin probe. Unfortunately, we did not find conditions that allowed us to both monitor actin filaments and achieve successful PB2 emission. Indeed, when the probe was sufficiently expressed to detect individual filaments, this was accompanied by major spindle rotation defects. From what we observed, we did not detect thick F-actin structure directly connecting the chromosomes or spindle to the cortex. Instead, we suspected the existence of a homogeneous cytoplasmic network of filaments merging with the cell cortex. This reminded us of the formin-2 assembled network already described in metaphase I oocytes (Azoury et al. 2008; Schuh and Ellenberg 2008). Therefore, one can speculate that this cytoplasmic network participates in transmitting F-Actin flows generated at the cortex to the rest of the cell. However, since we failed at imaging this network in satisfactory conditions, we did not include these considerations in the revised manuscript.

2/ Concerning the modeling, it is nice and simple, but maybe authors should clearly sate from the beginning that it is not quantitative and this is why all outcomes from simulations are presented in arbitrary units. Indeed, the intensities of two antagonistic forces applied on the spindle have not been measured, nor the viscosity of the cytoplasm, etc, etc…

Furthermore, one equation on spindle motion seems to be missing in the methods. It is not clear whether u_cyto, the ingression force, is computed on the center of the spindle (pivot point) or on both clusters of chromosomes. How also is the W deflection computed and whether it allows to keep a constant spindle length. The issue of spindle flexibility could be better explained.

Eventually, in the text (lane 292), it would be nice to explain to an audience of cell biologists where the noise comes from (rather than putting it in the methods section, lane 735). Indeed it is most certainly the noise which allows the breaking of symmetry so it is important to explain and discuss it in the main text.

**Comment 1.2**

We indeed tried to keep the model simple considering its qualitative nature. To underline this aspect, we have added few lines to the main text as follow: “Note that the use of an effective potential, added to the fact that physical quantities (forces, viscosity, etc) are not known quantitatively, make the model essentially qualitative and presented in arbitrary units. Its purpose is thus mainly to outline the fundamental mechanisms of symmetry breaking.”

See modifications in Results lines 307-311.

Concerning the spindle motion, no equation is missing from the methods, as the ingression is prescribed by the velocity of spindle mid-point (or central spindle). Therefore, it is not a force but a prescribed, constant velocity that defines the spindle motion in the model. To make that clear, we modified the relevant paragraph in the methods section: “The cytokinetic ingression acts as a pushing force on the spindle midpoint (corresponding to the central spindle). For simplicity, we assumed in the model that the ingression velocity is constant. Numerically, we simply prescribed the velocity of the central spindle, which moves with constant velocity $\vec{u}_{cyto}$ perpendicular to the spindle.”

See modifications in Methods lines 853-857.

To better explained the role of spindle rigidity and clarify the definition of the deflection w, we modified the relevant paragraph in the methods section: “It is because of the rigidity of the spindle (accounted for by the elastic restoring force), that the clusters follow the movement of the central spindle prescribed by $\vec{u}_{cyto}$.” ; “The deflection is equal to the distance between the mid-point of the (straight) line joining the two clusters and the mid-point of the spindle.”

See modifications in Methods lines 863-865.

Finally, as suggested, we also directly listed possible sources of noise in the main text: “Note that many sources of noise are possible in the biological system. These may include (but are not limited to): an asymmetry of the initial configuration, an asymmetry of the attraction forces, thermal or active fluctuations of the clusters’ position, an imperfect centering of the central spindle or of the ingression. Here, for the sake of simplicity, we only considered noise on the clusters’ position, although any source of noise would lead to symmetry breaking.”

See modifications in Results lines 320-324.

**Reviewer #2**

The perpendicular orientation of oocyte meiotic spindles at the cortex is thought to be essential for expulsion of the correct number of chromosomes into a polar body to prevent lethal aneuploidy. Thus the problem of meiosis II spindle rotation is highly significant. For mouse, the basic features of a single actin-dependent protrusion over the metaphase chromosomes that splits into two protrusions over the separating sets of anaphase chromosomes, then resolve into a single polar body, were described by Bernard Maro in 1984. This manuscript does not reference that paper.

**Comment 2.1**

Indeed, we referred to review articles and did not specifically mentioned Maro et al, 1984 and we apologize for that. We have now added the reference in the revised manuscript and have also inserted a sentence to summarize the results of Maro and colleagues in the introduction.

“As originally described by Maro and colleagues, this process is accompanied by the reorganization of the oocyte cortex with two F-actin-rich protrusions emerging above the spindle, before one of them becomes the PB2 while the other one retracts in the later stage of division”

See modifications in Introduction lines 66-69.

Throughout the 21st century, a number of labs have rediscovered actin-dependent meiotic spindle positioning in mouse oocytes and documented a number of molecules required but have not elucidated the actual mechanisms positioning the spindle. This manuscript uses modern microscopy methods to recapitulate Maro’s work and to demonstrate that several molecules already known to be active at metaphase, continue to be active during anaphase and spindle rotation. The manuscript does not elucidate the mechanism that moves one set of chromosomes toward the cortex during rotation which is the significant outstanding question. Using 3D projections of F-actin and myosin II light chain, the authors nicely show that one contractile ring over the metaphase chromosomes splits into 2 contractile rings, one over each set of anaphase chromosomes, then one protrusion relaxes as one set of chromosomes rotates into the stable protrusion. While very beautifully shown, the advance is somewhat incremental since one protrusion splitting into two protrusions that resolve again into one protrusion was described by Bernard Maro in 1984 (36 years ago).

**Comment 2.2**

In this commentary, the reviewer stated that our manuscript lacks novelty since “one protrusion splitting into two protrusions that resolve again into one protrusion” was already described by Maro and colleagues in 1984. We found this statement somewhat surprising since our study mostly focused on an aspect not covered in Maro et al, 1984, namely how symmetry breaking emerges during spindle rotation (which was clearly stated in the title and abstract of our manuscript). We addressed this question by providing, with Wang et al, 2020, the first live characterization of the rotation process and by monitoring the spindle position using an automated segmentation and tracking procedure. Two approaches that were, understandably, not possible in 1984.

Thus, it appears that the reviewer’s criticisms are more directed toward our results concerning the reorganization of cortical actomyosin following the oocyte activation (see Fig 3, Fig 4 and S4 Fig). In this regard, we would like to stress that our experiments, while agreeing with what has been described previously (Maro et al, 1984; Wang et al, 2011), provide new insights on how cortical actomyosin is chiefly regulated by the proximity of chromatids. Indeed, our data suggest that cortical F-actin tends to accumulate where RanGTP levels are the highest, whereas myosin-II reaches its maximum in the distal part of the gradient (Fig 3C and 3D). These simple rules, already suspected to be responsible for the polarization of the metaphase II oocyte (Deng et al, 2007), are enough to explain the variation of the cortical actomyosin pattern observed through the second meiotic division (see diagram in Fig 3E). Furthermore, we also demonstrated that two differentially regulated sub-populations of actomyosin co-exist in the polarized domain of activated oocytes (see Fig 3 and S4 Fig): a sub-population induced by chromatids/Ran/Cdc42 signalling and a sub-population induced by the central spindle/RhoA signalling. Note that none of these aspects (myosin-II localization, DNA-induced cortical actomyosin polarization, differentially regulated sub-populations) were addressed in the 1984 paper by Maro and colleagues.

Finally, our results concerning the cortical actomyosin reorganization were primarily intended to convince readers that the polarity system, that has been described to maintain the metaphase II spindle to the cell periphery (Yi et al, 2011), still functions after the anaphase onset. As such, these results should not be seen as the main findings of the paper, but rather as an intermediate step toward a better understanding of the mechanisms underlying symmetry breaking during spindle rotation.

This manuscript addresses a specific question common to all mitotic and meiotic spindle positioning situations. If a force moves both ends of the spindle toward the cortex, how does one end of the spindle pivot ahead of the other rather than becoming stalled with both spindle ends equidistant from the cortex. In mouse meiosis I spindle migration, micromanipulation was previously used to definitively show that the end of the spindle that is fortuitously closer to the cortex leads toward the cortex. In this manuscript, the authors use correlative observation and a mathematical model to suggest that the end of the spindle that is fortuitously closer to the cortex will lead during meiosis II rotation. By adding the assumption that velocity increases with proximity to the cortex, they can recapitulate rotation that requires stochastic noise in the initial parallel orientation. They do not do the micromanipulation experiment to demonstrate cause and effect. The result is thus not surprising because it is the same as the meiosis I result and it is not as rigorously demonstrated as the meiosis I result. The manuscript provides a very nice description of mouse meiosis II spindle rotation but does not provide a significant increase in mechanistic understanding as it is currently written. It is possible that the authors could improve the manuscript by explaining more clearly what is new.

**Comment 2.3**

From our perspective, events preceding spindle rotation are fairly different from those preceding the first polar body emission. Indeed, whereas at the onset of anaphase I the symmetry is already broken, with one pole of the spindle being clearly committed to the polar body (see perpendicular spindle/cortex configuration in the figure below), the anaphase II spindle must rotate in one direction before adopting a configuration compatible with polar body emission.

Therefore, it appears that the reviewer is comparing here the symmetry breaking events responsible for the metaphase I spindle migration with those responsible for the anaphase II spindle rotation. If some similarities are indeed remarkable, like the amplification of an initial asymmetry (common to all symmetry breaking events), we showed that the rotation of the anaphase II spindle involves distinct processes such as the contraction of the central spindle-induced cytokinesis ring (see Fig 2) and the separation of the polarized cortical domains (see Fig 3). We provide here an in-depth description of these processes that we believe are noteworthy.

Note that we will address the micromanipulation aspects in the Comments 2.5 of this document.

The mathematical model involves an inward force on the middle of the spindle generated by the ingressing actomyosin furrow (strong premise and molecular mechanism) and an “attractive force” on each set of separating chromosomes (weaker premise and no molecular mechanism). Rong Li’s lab suggested that cytoplasmic flow pushes the metaphase spindle toward the cortex but flow reverse during anaphase leaving no demonstrated model for the “attractive force”. The possibility that conventional dynein and microtubule-dependent pulling is involved, as occurs in C. elegans, appears to be dismissed while 2 publications reported rotation failure after partial microtubule depolymerization in rodent oocytes. This work offers only incremental advances unless they elucidate the mechanism of the attractive force.

Dominant negative cdc42 causes metaphase spindles to move away from the cortex at the same velocity as the cytoplasm flows away from the cortex. This is interpreted to mean that cortical cdc42 generates an attractive force that can resist cytoplasmic streaming. The molecular mechanism of this attractive force is not elucidated making the results highly descriptive rather than mechanistic.

**Comment 2.4**

As described in the Comment 1.1 of this document, we provided new experiments to reinforce our claims concerning the existence of attractive forces emanating from the polarized cortex. We also showed that these forces probably result from metaphase-like flows that persist during anaphase II. Although we agree that other factors could be implicated in these attraction forces, we do not think that microtubules, and more specifically the pulling of dynein motors on astral microtubules (current model in C. elegans), could be one of them. Indeed, the very dynamic behaviour of astral microtubules makes them particularly sensitive to microtubule depolymerizing agents. Thus, our nocodazole experiments (Fig 4) strongly suggest that DNA clusters remain attracted to the polarized cortex in the absence of astral microtubules. The figure below shows the lack of astral microtubules in low nocodazole-treated oocytes.

The investigators find that spindle rotation does not occur at a precise time relative to the extent of anaphase chromosome separation. This is an interesting observation supporting their idea that rotation is initiated by stochastic movement of one end of the spindle closer to the cortex.

During meiosis I spindle migration, the cortex proximal spindle pole leads and this has been demonstrated by micromanipulation. The current study does not use micromanipulation and thus only shows a correlation rather than cause and effect.

**Comment 2.5**

To address this comment, we assumed that the reviewer was referring to the study by Schuh and Ellenberg, 2008, in which the authors used microneedles to shift the metaphase I spindle position in order to modify the direction of its migration. Unfortunately, the way we perform oocyte imaging does not leave us much freedom to attempt micromanipulations. Indeed, we are using a small 37°C stage incubator (INUBG2E-GSI, Tokai Hit) whose enclosed configuration and restricted volume preclude the use of micromanipulation devices. That said, it would certainly be more difficult to implement such strategies during spindle rotation, as the symmetry-breaking events underlying this process occur more rapidly than during metaphase I spindle migration (~20 min vs. 2-3 hours). Indeed, the metaphase I spindle migration being a slow process, this leaves plenty of time to manipulate oocytes with microneedles and let them recover before resuming imaging.

However, using low dose of nocodazole, we managed to interfere with the normal course of spindle rotation (see Fig 4, S9 Movie and Comments 1.1 of this document). Our experiments showed that the fate of the normally internalized DNA cluster can be changed. Indeed, once detached from the anaphase spindle, the internalized cluster rapidly relocated to the cortex indicating that DNA clusters undergo attraction forces emanating from the polarized domains.

See modifications concerning nocodazole experiments in Results lines 227-244 and new Fig 5.

The authors should address whether ethanol-induced spindle rotation is the same as fertilization-dependent spindle rotation?

**Comment 2.6**

Ethanol has long been recognised as an efficient parthenogenetic activator to artificially trigger exit from metaphase-II arrest, protrusion of the second polar body, pronuclear formation, and even development to blastocyst in vitro (Cuthbertson, 1983; Surani et al., 1984; Dehapiot et al, 2013; Stein et al., 2020 for a recent review).

It is fair to ask whether the natural fertilization process triggers a similar spindle rotation for PB2 extrusion. To answer this, we performed in vitro fertilization (IVF) in HTF medium using fresh motile sperm obtained from OF1 males, capacitated in vitro for 3 hours in HTF. However, sperm binding to oocytes proved incompatible with live confocal imaging and automated 3D segmentation/tracking procedure. This was due to the rapid motility of the hyperactivated sperm flagellum that has made oocytes extremely shaky. In addition, zona pellucida-crossing by the sperm during natural fertilization is a lengthy process, making it extremely challenging to catch anaphase onset during live recordings. Instead, we performed IVF by inseminating cumulus-oocyte complexes, and fixed oocytes 1-3 hours after sperm addition. Oocytes were stained for F-actin (phalloidin) and spindle microtubules (anti-tubulin) to allow for a direct comparison with the data obtained in ethanol-activated oocytes.

As shown in the figure below, in vitro fertilized oocytes behaved similarly to ethanol-activated oocytes. Indeed, while the early anaphase configuration was essentially symmetrical, with two actin-rich protrusions developing over the segregating clusters of chromatids, the spindle later rotated to discard one cluster into the forming polar body. The spindle rotation was accompanied by the collapse of the other cortical protrusion and later followed by the cytokinesis ring closure at the base of the polar body. Note the presence of the paternal genetic material inside the fertilized oocytes.

These observations suggested that PB2 emission in fertilized oocytes is achieved through a similar sequence of events as seen with ethanol-activated oocytes. Therefore, we are confident that ethanol activation nicely recapitulates fertilization. However, since evidence for spindle rotation in fertilized mouse oocytes were already reported in earlier studies (Maro et al., 1984; Zhu et al., 2003; Larson et al., 2010), we felt it was not necessary to incorporate these data in the manuscript. Please let us know if you think otherwise.

Fig. S1 demonstrating reversal of cortical flow at anaphase onset appears to be a single example and the average result from many oocytes is not presented.

**Comment 2.7**

To be more systematic in our PIV measurements, we developed a new procedure to monitor and compare cytoplasmic streaming patterns between oocytes and conditions. To do so, we first selected oocytes whose 3D orientation was compatible with PIV and registered them in space and time before running the analysis. We then averaged the vector fields to obtain flow maps representative of the experimental conditions. Two examples of these maps, comparing metaphase II and anaphase II oocytes populations, can now be seen in S1E Fig. In parallel, we also measured the reversion of cytoplasmic streaming over time by restricting our PIV analysis to the central part of oocytes (see PIV ROI in S1B Fig) and extracting velocity vectors components along the axis defined by the polarized domain and the opposite side of the cell. This enabled us to produce temporal curves whose positive values indicated a flow oriented toward the oocyte centre (inverted flow) and negative values a flow oriented toward the polarized domain (metaphase-like flow). By doing so, we observed that the reversion of cytoplasmic streaming generally peaks 20 minutes after oocyte activation (see new S1F Fig).

See new PIV procedure in Results lines 102-110, Methods lines 820-837, and new S1 Fig.

Line 104: The direction or nature of the “distinct flows” occurring at the time of protrusion collapse and ring closure are not described and the word “generated” should be replaced with “correlated in time” since no cause and effect relationship is demonstrated.

**Comment 2.8**

Done, see modifications in Results lines 109-110.

What are the particles being tracked in the PIV in figure S1 and what is the evidence that velocities represent the same particle that remains in focus for the duration of the velocity measurement?

**Comment 2.9**

Particle image velocimetry does not rely on tracking individual particles but rather measures the mean displacement of groups of contrasted particles/objects over time. For this, PIV algorithms split images into smaller interrogation windows and compute maximum 2D cross-correlations to extract local displacement vectors. PIV algorithms are known to be easy to implement and fast to compute as compare to individual particle tracking techniques. However, PIV measurements cannot capture local diffusive behaviour and lose accuracy if the particle flow occurs over a contrasted static background. These limitations were not a problem for us since we mostly measured coherent flows carrying all cytoplasmic particles at the cell scale. Also, the fact that our manual particle tracking in Fig 4 was consistent with the PIV vector field further validated this approach.

Line 134: Localization of ECT2, anillin and rho kinase is referenced to Fig. S1 but this data is in Fig. S2. This data needs to be quantified. How many oocytes exhibited the pattern shown? How many did not exhibit the pattern shown?

**Comment 2.10**

Correct mislabelled figure

Done, see new S3 Fig.

Quantitation

Done, see modifications in S3 Fig legend lines 484-486.

It would be more of an advance to show co-localization of rhoA with F-actin and myosin II and localization of rhoA in latrunculin treated oocytes so that the reader can see whether rhoA simply co-localizes with F-actin or if there is a cause-effect relationship.

**Comment 2.11**

Our data concerning the RhoA accumulation in the cortical region facing the central spindle have been obtained using trichloroacetic acid (TCA) as a fixative since we did not achieve reliable RhoA detection using paraformaldehyde (PFA). Unfortunately, TCA fixation precludes the detection of actin filaments and we therefore aimed for a different strategy to detect both RhoA and the F-Actin. We expressed the highly specific RhoA-GTP biosensor eGFP-AHPH generated by the Glotzer lab, which is based on the RhoA-binding domain of anillin (Piekny and Glotzer, 2008). We then fixed oocytes using PFA and observed a distinct accumulation of active RhoA in the cytokinetic furrow facing the central spindle (S4A and S4B Fig). This accumulation co-localized with the central spindle-facing F-Actin patch, which itself co-localized with the central spindle-facing myosin-II (Fig 3A).

To examine a possible cause-effect relationship, we treated oocytes with Latrunculin A, as suggested, in order to disassemble F-actin. As expected, Latrunculin A (2 µM) induced a substantial loss of actin filaments in the oocyte cortex, and in particular, a complete loss of the central spindle-facing F-actin patch, associated with the absence of furrow ingression (S4A and S4B Fig). Remarkably, eGFP-AHPH remained associated with the cortical area overlying the central spindle, indicating that the localized activation of RhoA was unaffected. Therefore, we concluded that actin filament nucleation (via the central spindle pathway) is not a prerequisite for RhoA activation and localization. Our data suggested instead that RhoA lies upstream of actin polymerization in the cytokinesis furrow (patch), because treatment with Bi-2536 to inhibit ECT2 recruitment at the central spindle also led to a loss of the F-actin patch (S4C and S4D Fig).

See modifications in Results lines 183-192, and new S4 Fig.

Line 142: The authors should clarify whether the quantitation presented for polar body extrusion also applies to the failure to localize rhoA and the failure in ingression.

**Comment 2.12**

The quantitations concerning polar body extrusion (previously Fig 2C histogram) are not related to images illustrating failure of RhoA localization and ingression (previously Fig 2C images). To avoid further confusion, we separated the panel (see new Fig 2C and 2D) and added quantitation for images of new Fig 2C in the figure legend.

See modifications in Fig 2 and Fig 2 legend lines 464-468.

Quantitation of the number of oocytes exhibiting the flow pattern in Fig. 2G is needed.

**Comment 2.13**

As described in Method, Comment 2.5 and 3.3 of this document, we developed a new procedure to be more systematic in our PIV measurements. Indeed, while in the first version of the manuscript we simply showed one representative example of the flow observed upon Bi-2536 inhibition, we now show an averaged flow map of 32 control and 13 Bi-2536 treated oocytes.

See modifications in Fig2 legend lines 476-478.

The finding that a Plk1 inhibitor prevents rhoA localization, ingression and rotation is interesting. However, Plk1 is involved in many different pathways. The authors should at least show correct localization of MgcRacGAP and localization failure for ECT2 in response to the Plk1 inhibitor if the results are to be related to the pathway diagrammed in Fig. 1A.

**Comment 2.14**

These experiments are indeed judicious to substantiate our claims on the effects of PLK1 inhibition. We therefore performed immuno-staining against MgcRacGAP and monitored the localization of the eGFP-ECT2N fusion construct in Bi-2536 treated oocytes.

Correct localization of MgcRacGAP

Using a commercial antibody, we detected MgcRacGAP at the midline of the central spindle in anaphase II (S3B Fig). This is consistent with the canonical central spindle pathway described in Fig 2A, whereby MgcRacGAP, in association with MKLP1, form the so-called centralspindlin complex, which accumulates at the spindle midzone (Basant and Glotzer, 2018). In oocytes treated with Bi-2536, we also observed an accumulation of MgcRacGAP at the central spindle midzone, demonstrating that the correct localization of MgcRacGAP was unaffected by PLK1 inhibition. Note however that the intensity of MgcRacGAP staining was increased in PLK1-inhibited oocytes. We speculate that this could result from epitope unmasking, due to the fact that the MgcRacGAP antibody was raised against an internal fragment that encompasses the PLK1 phosphorylation sites. The loss of the bulky phosphate groups, associated with the release of bound ECT2, may have facilitated antibody access to its epitope, in Bi-2536 treated oocytes.

Localization failure for ECT2

To monitor ECT2 localization, we first expressed an eGFP-fusion of the full-length mouse ECT2 protein. While the localization of eGFP-ECT2 at the central spindle was readily detected, oocytes expressing the fusion protein were severely deformed. We assumed that overexpressing ECT2 may have led to ectopic Rho GTPase activation, leading to excessive actomyosin contractility. To avoid this cell deformation, we opted for a strategy previously described by the Miki and Glotzer labs: we expressed instead the NH2-terminal half (aa 1-452) of ECT2, devoid of the catalytic GEF domain and encompassing the two BRCT domains (eGFP-ECT2N), which is sufficient for MgcRacGAP recognition and localization to the central spindle in a PLK1-dependent manner (Tatsumoto et al., 1999; Wolfe et al., 2009). Using this tool as a reporter for ECT2 localization, we could demonstrate that PLK1 inhibition with Bi-2536 effectively prevented ECT2 localization to the central spindle (S3 Fig).

In this regard, we must admit that we made a mistake in the first version of the manuscript. Indeed, the eGFP-ECT2 images (metaphase II and anaphase II) presented in previously S2 Fig (now in S3A Fig) have been obtained by expressing this truncated version of ECT2 (eGFP-ECT2N) and not the full-length version. This has now been corrected to eGFP-ECT2N.

See modifications new experiments in Results lines 145-151 and new S3 Fig.

Line 157: Do the authors mean “carried by the metaphase II spindle”?

**Comment 2.15**

Done, see modifications in Results lines 167.

Fig.3A: The third column should be labeled (presumably pMLC2).

**Comment 2.16**

Done, see modifications in new Fig 3.

Line 176 and Fig S3 need quantitation. How many oocytes exhibited this result and how many did not?

**Comment 2.17**

Done, see modifications in S4 Fig legend lines 536-538.

Line 240: “we measured a speed of about 0.3 μm.min-1,” must be replaced with a mean, sem and n.

**Comment 2.18**

As described in Method, Comment 2.5 and 3.3 of this document, we developed a new procedure to be more systematic in our PIV measurements. Indeed, while in the first version of the manuscript we simply showed one representative example of the flow observed upon Cdc42 inhibition, we now show averaged flow maps of 32 control and 29 Cdc42T17N oocytes. The sentence has been modified accordingly.

Done, see modifications in Results lines 276-279.

**Reviewer #3**

The present study provides new insights into the rotation of meiotic spindle in mouse meiosis - a process that has been largely overlooked in mouse oocytes and its precise mechanisms have remained elusive. By having established a quantitative and systematic approach in live oocytes, the authors provide some evidence that spindle rotation may be directly controlled by membrane invagination during cytokinesis/polar body extrusion, thereby supporting conclusions of previous studies that have linked spindle rotation and membrane invagination (e.g. Wang et al, 2011). In addition, antagonizing these inward forces exerted on the spindle, the authors propose the existence of attractive outward forces acting on the spindle. The authors do not provide direct evidence for the existence of these outward forces, nor their underlying mechanisms. Instead the authors use mathematical modelling predicting the existence of these forces to reconstitute aspects of spindle rotation. Overall, a thorough study, which requires some more direct evidence to support the authors' hypotheses.

- In Figure 2, the authors use a PKL1 inhibitor to block cytokinesis in oocytes, which subsequently fail to undergo spindle rotation. However, spindles do not only fail to rotate, but also start spinning around along the cell periphery as mechanisms required to maintain the spindle in its position are inhibited. This leaves the question open whether spindle rotation fails because of cytokinetic failure or loss of anchorage mechanisms. It could be argued that loss of spindle position alone is sufficient to hinder the establishment of the cytokinetic machinery above the spindle. Thus to strengthen the authors' conclusion that cytokinetic ring assembly and cortical ingression drives spindle rotation could benefit from additional evidence. For example, could the authors target more directly components of the cytokinetic machinery such as ECT2, Rho or Rock-1, by using specific drugs or dominant-negative variants? It would be helpful if under these conditions the spindle remains in place and solely cytokinesis is perturbed.

**Comment 3.1**

In the first version of the manuscript, we only showed a spinning spindle oocyte to illustrate the PLK1 inhibition phenotype. This was somewhat misleading since in fact the majority of Bi-2536 treated spindles simply remained parallel to the cortex. Indeed, by measuring the angle formed by oocyte centroid and spindle mid-points at the beginning and the end of rotation, we observed that only one third of the Bi-2536 treated spindles underwent spins superior to the regular displacement observed in control oocytes (see S3C Fig). This suggested that the lack of spindle rotation was indeed due to cytokinetic failure and not a loss of anchorage mechanisms. To avoid further confusion, we added a non-spinning Bi-2536 treated spindle to the Fig 2E and S7 Movie, and showed the spin angle quantifications in S3C Fig. As suggested, we also measured the spin angle in Rock-1 inhibited oocytes (see Y-27632 condition in the figure below). However, as we did not observe a difference with the Bi-2536 treated oocytes (both showing a similar proportion of spinning spindle) we did not include this condition in the new version of the manuscript. If you think otherwise, please do let us know.

See modifications in Results lines 152-154, and new Fig 2 and S3 Fig.

- Fig. 5D: In oocytes with dominant-negative Cdc42 that are still able to undergo cytokinesis, despite a small polar body (group 1), still appear to rotate their spindle successfully and effectively. However, from the authors' initial data one would expect that a perturbed polar body extrusion should somewhat impact on spindle rotation. In these cells, is spindle rotation delayed?

**Comment 3.2**

To answer this question, we measured the initial time of rotation (ti rotation) in Cdc42T17N injected oocytes showing a small polar body phenotype. We observed that, under this condition, the rotation is initiated later (26.0 vs. 20.7 min) and is slightly longer to complete (58.0 min vs. 50.4 min). This suggest that, indeed, the overall spindle rotation dynamics is perturbed under this inhibitory condition. However, because these results proved to be hardly statistically significant, we have not incorporated them into the manuscript. If you think otherwise, please do let us know.

- Fig. 5G: These data require more thorough explanation. The authors show that the inward-directed cytoplasmic flows and spindle off-centering in dominant-negative Cdc42 occur at the same speed. The authors then conclude that the spindle off-centering is because the Cdc42 creates counterforces at the spindle to resist this inward flow. Alternatively, one could speculate that the cytoplasmic streaming forces are increased in dominant-negative Cdc42 resulting in spindle off-centering. Thus, the authors should clarify whether the flow velocities have been measured in dominant-negative Cdc42 and/or in control oocytes. They also could provide data to compare the flow velocities in control and Cdc42 to strengthen their conclusion that it is not an increase in inward-directed flows that leads to spindle off-centering.

**Comment 3.3**

In response to this comment, we conducted a more thorough analysis of the cytoplasmic streaming occurring in both control and Cdc42T17N injected oocytes. We first produced averaged PIV maps (Fig 6F) and temporal flow curves (S5C Fig) following the procedures described in Method and Comment 2.5 of this document. We next measured maximum flow velocities (average of 10 % max. values, S5C-D Fig) and spindle relocalization speeds (slope of linear fits, S5E-F Fig) during an analysis window extending from 10 to 50 minutes after activation. This allowed us to compare these two quantities, oocyte by oocyte, and show that they are clearly correlated (S5G Fig).

On the other hand, our measurements also showed that, on average, the Cdc42T17N injected oocytes, whose spindle relocated to the centre of the cell (Reloc), underwent stronger flow reversal than those extruding a small polar body (sPB) (S5D Fig). This, as hypothesised by the reviewer, suggests that an increased flow velocity, and not a lack of attracting forces, could explain the spindle relocation phenotype. However, we observed that many control oocytes displayed flow velocities as high as those measured in spindle relocating Cdc42T17N oocytes. This suggests that the strength of the flow alone is unlikely to explain the relocation phenotype. We stand for the idea that attraction forces, exerted by the polarized F-Actin cortex (see Fig 6), play a critical role in maintaining the spindle at the cell periphery during anaphase II.

See modifications in Results lines 269-282, and new Fig 6 and S5 Fig.

- In line with these experiments, the authors conclude on line 248/249 that their Cdc42 loss of function experiments showed that the polarized domain exerts attractive forces on the chromosomes. However, the existence of such forces can at best be suggested from the provided experiments but are not demonstrated. To strengthen their conclusion, could the authors provide direct evidence for such forces by acutely inhibiting key players (Arp2/3, WASP or Myosin-II) in anaphase II that are involved in attracting the spindle in metaphase II? Alternatively, overexpression of downstream factors such as Arp2/3, WASP etc. might rescue to the spindle off-centering defects in dominant-negative Cdc42 oocytes. The authors' conclusion would greatly benefit from such and other experiments that show more directly the existence of such attractive forces acting on the spindle.

- A previous study (Yi et al, 2011) showed that upon loss of Arp2/3 the direction of cytoplasmic streaming is reversed in metaphase II and that this inversion might depend on Myosin-II function. Thus it is logical to speculate that Myosin-II might also be responsible for the detected inward flows in anaphase II. Have the authors tested the effects of Myosin-II inhibition on cytoplasmic flows in control and dominant-negative Cdc42, as well as on spindle off-centering in dominant-negative Cdc42?

**Comment 3.4**

To address these points, we first sought to reproduce the Cdc42T17N spindle relocation phenotype using an alternative approach. We decided to target the most downstream effector of Cdc42, the Arp2/3 complex, which is responsible for polarizing the F-Actin cortex (Sun et al, 2011; Yi et al, 2011). Thus, we treated oocytes with the CK-666 inhibitor and fixed them 90 min after activation. We succeeded to nicely reproduce the spindle relocation phenotype, as most of the treated oocytes relocated their spindle to the centre of the cell (Fig 6D). These experiments validated the spindle relocation phenotype observed upon Cdc42 inhibition and further indicated that this effect was due to the cortical F-Actin polarization activity of the GTPase. Furthermore, since the CK-666 has previously been shown to prevent the emergence of metaphase-like flows in both metaphase I and II (Yi et al, 2013; Yi et al, 2011) this also reinforced the idea that these flows are responsible for attracting the chromosomes to the cortical vicinity (see metaphase-like flows in Fig 4).

Secondly, we also sought to rescue the Cdc42T17N spindle relocation phenotype by preventing the cytokinetic contraction. For this, we targeted the myosin-II of the cytokinesis ring using the Rock-1 inhibitor, Y-27632. Note that the myosin-II of the cytokinesis ring is differentially regulated from the myosin-II rings surrounding the polarized domains. Indeed, while the former is promoted by the central spindle pathway (ECT2-RhoA-Rock-1), the latter depends on chromosome-centered Ran-Cdc42 signalling (see Fig 3 and S4 Fig). The Y-27632 treatment resulted in a complete loss of cytoplasmic streaming in Cdc42T17N oocytes (Fig 6E, 6F and S11 Movie) and rescued the spindle relocation phenotype in both Cdc42T17N and CK-666 treated oocytes (Fig 6C and 6D). This confirmed that inverted cytoplasmic streaming is indeed responsible for the spindle relocation phenotype.

See modifications in Results lines 254-290, and new Fig 6.

**Minor points:**

- Fig. 4C: It appears that only individual cells are represented in the graphs, but better to show mean of several cells, if possible. In fact, Fig, 4C suggests that the outward cluster becomes attracted to the cell periphery (and inward cluster repelled from the cell periphery) before the onset of spindle rotation (about 20 minutes). Could the authors comment on this asymmetry is achieved before spindle rotation?

**Comment 3.5**

In fact, we extensively address this point in the last figure of the manuscript. Indeed, we looked at the variation over time of the averaged DNA cluster distance to the cortex (see middle panel in Fig 7E) and the DNA cluster distance to the cortex ratio before ti rotation (Fig 7F). We observed that, indeed, the externalized DNA cluster tended to be closer to the cortex before the onset of spindle rotation. Furthermore, we showed that a higher initial asymmetry resulted in a faster initiation of rotation (Fig 7G).

These aspects are commented in Results lines 300-359, and new Fig 7.

- Line 236: Speaking of inversion of flows, is confusing here, as in the given context this implies that the flows are inverted from control to dominant-negative Cdc42 oocytes. If authors meant to say that the flows were inverted from metaphase II to anaphase II, this should be stated more clearly.

**Comment 3.6**

We modified the text to avoid confusion, see modifications in Results lines 269-272.

- Line 240, Fig. 5G: Can the authors specify how the average speed of cytoplasmic flows was measured - only in the central part of the cell or as an average of the entire cell? Also, it should be clearly stated from how many cells these were measured. It appears that in Fig. 5G, only one cell per condition was used to perform vector measurements. If possible, could authors provide an overlay of several cells e.g. in form of a heatmap?

**Comment 3.7**

As described in Method, Comment 2.7 and 3.3 of this document, we developed a new procedure to be more systematic in our PIV measurements. Indeed, while in the first version of the manuscript we simply estimated the average flow velocity by examining vector fields, we have now quantified flow velocities in individual oocytes. This was made by measuring flow velocities in the central part of the oocyte (see PIV ROI in S1B Fig) along an axis defined by the polarized cortical domain and the opposite part of the cell. Also, as suggested by this reviewer, we have generated averaged flow maps to compare cytoplasmic patterns between conditions (see Fig 6F but also S1E Fig and Fig 2H).

See new PIV flow maps in new Fig 6 as well as the new PIV procedure in Results lines 102-110, Methods lines 820-837, and new S1 Fig.

**Reference**

Azoury J, Lee KW, Georget V, Rassinier P, Leader B, Verlhac MH. Spindle positioning in mouse oocytes relies on a dynamic meshwork of actin filaments. Curr Biol. 2008 Oct 14;18(19):1514-9.

Schuh M, Ellenberg J. A New Model for Asymmetric Spindle Positioning in Mouse Oocytes. Curr Biol. 2008;18(24):1986–92.

Maro B, Johnson MH, Pickering SJ, Flach G. Changes in actin distribution during fertilization of the mouse egg. J Embryol Exp Morphol. 1984 Jun;81:211-37.

Wang H, Li Y, Yang J, Duan X, Kalab P, Sun SX, et al. Symmetry breaking in hydrodynamic forces drives meiotic spindle rotation in mammalian oocytes. Sci Adv. 2020 Apr3;6(14):eaaz5004.

Wang Q, Racowsky C, Deng M. Mechanism of the chromosome-induced polar body extrusion in mouse eggs. Cell Div. 2011;6:1–9.

Deng M, Suraneni P, Schultz RM, Li R. The Ran GTPase Mediates Chromatin Signaling to Control Cortical Polarity during Polar Body Extrusion in Mouse Oocytes. Dev Cell. 2007 Feb;12(2):301–8.

Yi K, Unruh JR, Deng M, Slaughter BD, Rubinstein B, Li R. Dynamic maintenance of asymmetric meiotic spindle position through Arp2/3-complex-driven cytoplasmic streaming in mouse oocytes. Nat Cell Biol. 2011 Oct 28;13(10):1252–8.

Cuthbertson KS (1983). Parthenogenetic activation of mouse oocytes in vitro with ethanol and benzyl alcohol. J. Exp. Zool. 226 : 311-314.

Surani MAH, Barton SC and Norris ML (1984). Development of reconstituted mouse eggs suggests imprinting of the genome during gametogenesis. Nature 308 : 548-550.

Dehapiot B, Carrière V, Carroll J, Halet G. Polarized Cdc42 activation promotes polar body protrusion and asymmetric division in mouse oocytes. Dev Biol. 2013;377(1):202–12.

Stein P, Savy V, Williams AM and Williams CJ (2020). Modulators of calcium signalling at fertilization. Open Biol. 10 : 200118.

Zhu ZY, Chen DY, Li JS, Lian L, Lei L, Han ZM, et al. Rotation of meiotic spindle is controlled by microfilaments in mouse oocytes. Biol Reprod. 2003;68(3):943–6.

Larson SM, Lee HJ, Hung PH, Matthews LM, Robinson DN and Evans JP (2010). Cortical mechanics and meiosis II completion in mammalian oocytes are mediated by myosin-II and Ezrin-Radixin-Moesin (ERM) proteins. Mol. Biol. Cell. 21 : 3182-3192.

Piekny AJ and Glotzer M. Anillin is a scaffold protein that links RhoA, actin, and myosin during cytokinesis. Curr. Biol. 2008; 18:30-36.

Basant A, Glotzer M. Spatiotemporal Regulation of RhoA during Cytokinesis. Curr Biol. 2018;28(9):R570–80.

Tatsumoto T, Xie X, Blumenthal R, Okamoto I, Miki T. Human ECT2 Is an Exchange Factor for Rho GTPases, Phosphorylated in G2/M Phases, and Involved in Cytokinesis. Cell. 1999;147(5):921–7.

Wolfe BA, Takaki T, Petronczki M, Glotzer M. Polo-like kinase 1 directs assembly of the HsCyk-4 RhoGAP/ECT2 RhoGEF complex to initiate cleavage furrow formation. PLoS Biol. 2009 May 5;7(5):e1000110.

Sun SC, Wang ZB, Xu YN, Lee SE, Cui XS, Kim NH. Arp2/3 complex regulates asymmetric division and cytokinesis in mouse oocytes. PLoS One. 2011 Apr 8;6(4):e18392.

Yi K, Rubinstein B, Unruh JR, Guo F, Slaughter BD, Li R. Sequential actin-based pushing forces drive meiosis I chromosome migration and symmetry breaking in oocytes. J Cell Biol. 2013;200(5):567–76.
